# CD40 Signaling Synergizes with TLR-2 in the BCR Independent Activation of Resting B Cells

**DOI:** 10.1371/journal.pone.0020651

**Published:** 2011-06-02

**Authors:** Shweta Jain, Sathi Babu Chodisetti, Javed N. Agrewala

**Affiliations:** Immunology Laboratory, Institute of Microbial Technology, Council of Scientific and Industrial Research, Chandigarh, India; Universita di Sassari, Italy

## Abstract

Conventionally, signaling through BCR initiates sequence of events necessary for activation and differentiation of B cells. We report an alternative approach, independent of BCR, for stimulating resting B (RB) cells, by involving TLR-2 and CD40 - molecules crucial for innate and adaptive immunity. CD40 triggering of TLR-2 stimulated RB cells significantly augments their activation, proliferation and differentiation. It also substantially ameliorates the calcium flux, antigen uptake capacity and ability of B cells to activate T cells. The survival of RB cells was improved and it increases the number of cells expressing activation induced deaminase (AID), signifying class switch recombination (CSR). Further, we also observed increased activation rate and decreased threshold period required for optimum stimulation of RB cells. These results corroborate well with microarray gene expression data. This study provides novel insights into coordination between the molecules of innate and adaptive immunity in activating B cells, in a BCR independent manner. This strategy can be exploited to design vaccines to bolster B cell activation and antigen presenting efficiency, leading to faster and better immune response.

## Introduction

Stimulation of B cells through antigen specific B cell receptor (BCR) leads to their activation, proliferation and differentiation to antibody secreting plasma cells. Besides BCR, B cells also express an array of molecules that assist in regulating both innate and adaptive immune responses. Such examples include costimulatory molecules involved in adaptive immunity and Toll like receptors (TLRs) responsible for innate immunity [1], [2]. It is well established that co-engagement of BCR with these accessory molecules lead to heightened B cell response. For example, synergism between BCR and TLRs augments expression of NF-κB, MAPK p38, leading to enhanced B cell activation, proliferation and differentiation [3]–[5].

Recently, many reports have highlighted the role of costimulatory molecules such as CD40, CD80 and CD86 in not only influencing T cells but also B cells through bidirectional signaling [6]–[8]. Among all costimulatory molecules expressed on B cells, CD40 is extremely important due to its role in assisting the activation, proliferation, differentiation, survival and generation of memory B cells [9], [10]. Further, studies on CD40^−/−^ mice have established that such B cells failed to proliferate and undergo isotype switching [11]–[13].

TLRs, on the other hand, are germline encoded molecules that are virtually expressed on all cells of immune system. They are a family of Pattern Recognition Receptors (PRRs) that recognize conserved motifs called Pathogen Associated Molecular Patterns (PAMPs) on the surface of microbes [2]. Binding of PAMPs with TLRs affects the functions of antigen presenting cells (APCs). For example, signaling through TLRs leads to the expression of costimulatory molecules on B cells, dendritic cells (DCs), macrophages, etc. [14]–[16]. Most TLRs such as TLR-2, 3, 4, 7 and 9 have been implicated in modulating B cell response. Among all TLRs, TLR-2 is considered quite critical molecule of innate immunity that regulates humoral immunity [15], [17]–[19].

Evidences indicate that B cells can also be activated through alternative pathways independent of BCR [8], [20], [21]. Moreover, nothing has been very precisely documented indicating the concerted role of costimulatory molecules and TLRs in regulating the activation of resting B (RB) cells. Hence, in the present study, we investigated whether triggering through costimulatory molecules can modulate the activity of B cells stimulated through TLRs. For this, we tried various combinations of costimulatory molecules CD40, CD80 and CD86 in conjunction with TLR-2, TLR-4 and TLR-9. Interestingly, we observed that cross-linking of CD40 significantly bolsters the activation, proliferation, differentiation, calcium flux, antigen uptake and ability to help CD4 T cells of TLR-2 stimulated RB cells.

## Results

### Signaling through CD40 augments proliferation of TLR-2 stimulated RB cells

**Figure 1:**
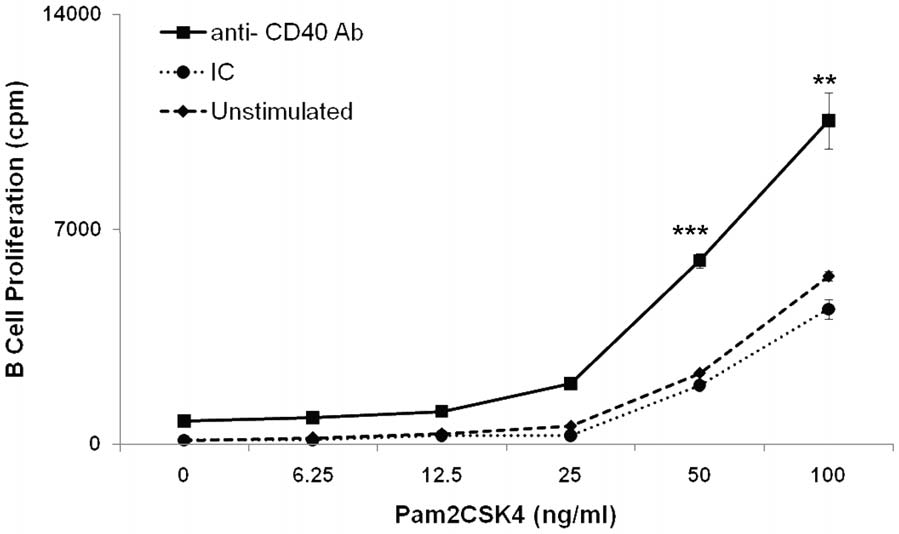
CD40 costimulation augments the proliferation of TLR-2 stimulated RB cells. RB cells were stimulated with anti-CD40 Ab and Pam2CSK4 (TLR2.CD40) and cultured for 16 h. ^3^H thymidine (0.5 µCi/well) was added and proliferation was checked after 16 h by liquid scintillation counting. Data indicate counts per min (cpm) and expressed as mean ± SEM of triplicate wells. Results are representative of four independent experiments. ‘*’, ‘**’, ‘***’ indicates p<0.05, p<0.01, p<0.001 respectively.

First we examined whether signaling through TLR-2 can render RB cells responsive to CD40 costimulation. This phenomenon was seen in a dose-responsive manner in cells stimulated through both TLR-2 and CD40 (TLR2.CD40) (Fig. 1). Maximum proliferation was achieved with 100 ng/ml of TLR-2 agonist Pam2CSK4 when used in combination with 0.5 µg of anti-CD40 Ab for triggering. We also noticed that Pam2CSK4 alone (100 ng/ml), in the absence of CD40 triggering also induced proliferation but the magnitude was significantly (p<0.01) lesser when compared with TLR2.CD40 activated RB cells. Further, the extent of B cell proliferation noticed with Pam2CSK4 (100 ng/ml) alone, could be achieved with half the concentration (50 ng/ml) of Pam2CSK4 when acting in conjunction with CD40 signaling (Fig. 1). We further substantiated this finding with microarray data (Table S1). We found that TLR2.CD40 activated RB cells upregulated the expression of genes encoding TNF receptor super family member Tnfrsf13b, which plays an important role in B cell activation and differentiation. Upregulated expression of Cd81 is also indicative of augmented CD81 mediated CD19 signalosome activity, which plays a key role in the regulation of B cell development, activation, growth and motility. This complex reduces the threshold for B cell activation via the BCR by bridging antigen specific and CD21-mediated complement recognition [22]. In contrast, signaling through TLR2.CD40 downregulated expression of caspase-3 which is involved in apoptosis. Downregulation was also observed in gene Inpp5d, which is a phosphatidytlinositol phosphatase involved in negative regulation of BCR signaling [23]. Consistent with this, there was also decreased expression of gene encoding Fc-gamma-RIIB (FCGR2B) receptor whose signals are mediated through Inpp5d. FCGR2B plays a central role in terminating signal transduction from activated immune complexes and acts as a negative regulator of proliferation [24], [25]. These observations gave adequate convincing indications that costimulation through CD40 can effectively induce the proliferation of TLR-2 stimulated RB cells.

### TLR2.CD40 stimulated RB cells display activation phenotype

**Figure 2:**
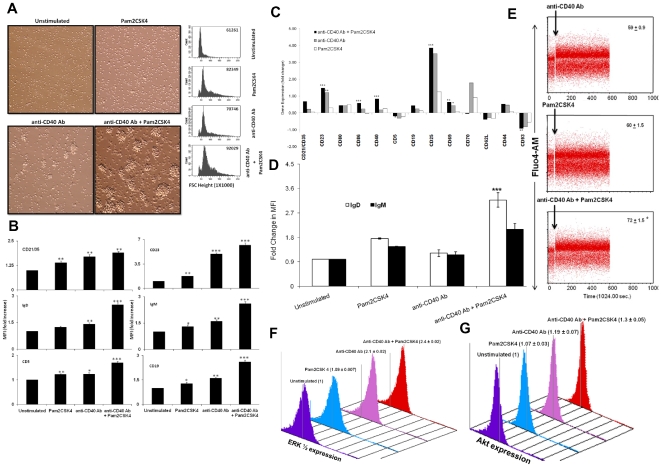
Signaling through CD40 bolsters the activation phenotype of the TLR-2 stimulated RB cells. (A) Signaling was delivered in RB cells with Pam2CSK4 and anti-CD40 Ab for 16 h and formation of B cell blasts (left panel) was seen microscopically by bright field images (20×1.6× magnification). Shown here are the representative images from 5–6 fields from each culture well. Change in forward scatter was assessed through flow cytometry (right panel). X-axis depicts increase in size of B cells (forward scatter, FSC; 1×1000) on a linear scale. The FSC values in inset are normalized with isotype-matched control. (B) Stimulated B cells were stained for the expression of B cell activation markers CD21/35, CD23, CD19, IgM, IgD and CD5. Fold change in mean fluorescence intensity (MFI) was calculated with respect to unstimulated controls. Data are pooled from 3–6 independent experiments and expressed as average fold change (mean ± SEM) in the expression with respect to unstimulated controls. (C) Modulation in the expression of genes involved in B cell activation as analysed through microarray. Bars indicate fold change in geometric mean of the gene expression. Data is representative of one of the biological replicate experiments. (D) Surface expression of IgD and IgM on stimulated RB cells. Bars indicate average fold change (mean ± SEM) in the expression of IgD and IgM. Data are pooled from 3–6 independent experiments and expressed as average fold change (mean ± SEM) in the expression with respect to unstimulated controls. (E) TLR2.CD40 stimulated RB cells were loaded with Fluo-4 AM dye and calcium flux was measured over a period of 10 minutes. Shown here are representative dot plots from 3 independent experiments. Values in the inset are the averaged geometric mean of MFI of Fluo-4 AM from three experiments with SEM. (F,G) TLR2.CD40 stimulated cells were stained for intracellular phosphorylated ERK1/2 and Akt and analysed by flow cytometry. Shown here are plots from one of the three independent experiments and values represent mean ± SEM of fold change in expression with respect to unstimulated controls. . ‘*’, ‘**’, ‘***’ indicates p<0.05, p<0.01, p<0.001 respectively.

We next enquired whether the TLR2.CD40 stimulation of RB cells results in augmented expression of costimulatory molecules CD80, CD86 and CD40. Remarkably, the expression of CD40, CD86 and CD80 was significantly enhanced (Fig. S1, A,B). Similarly, enhancement in the expression of MHC and TLR-2 was also noticed (Fig. S1, C). We also observed an appreciable improvement in the size and blast formation on CD40 costimulation of TLR-2 elicited RB cells (Fig. 2A). Further, upregulation of the activation markers, such as CD21/35, CD23, IgD, IgM, CD5 and CD19 was also noticed (Fig. 2B, Fig. S2 A) [26]–[32]. We found that signaling through TLR2.CD40 not only modulates the activation profile of the effector molecules (IgM, IgD, CD5, CD23, CD19) but also leads to conglomeration of other molecules (CD21/35), which are thought to play an important role in linking innate and adaptive immunity (Fig. 2B, Fig. S2 A). A moderate upregulation in the expression of CD5 (a negative regulator of B cell activation) and dramatic increase in CD19 expression (a positive regulator of B cell activation) indicated a balance between two opposing phenomenon (tolerance and activation) (Fig. 2B, Fig. S2 A). This event may be crucial in maintaining cellular and physiological homeostasis. This observation was also supported by gene profiling of the molecules involved in the B cell activation (Fig. 2C). We also noted a significant increase in the gene expression of Cd23 (p = 0.002), Cd86 (p = 0.0002), Cd40 (p = 0.0039) and Cd19 (p = 0.003), indicating an activated phenotype. We also found that TLR2.CD40 signaling down regulates CD93 more significantly, as compared individually. Moreover, Cd25 and Cd69 are also augmented, giving an indication of activated phenotype (Fig. 2C).

Activated B cells always exhibit 5–10 folds higher IgD than IgM, but as the activation progresses, the expression profile shifts more towards IgD [33], [34]. We observed that TLR2.CD40 stimulated RB cells upregulated IgD and IgM expression (Fig. 2D, Fig. S2 B) but the relative increase in IgD was far more as compared to IgM. While IgD expression was enhanced (MFI: 12251) on stimulation with TLR2.CD40 as compared to CD40 alone (MFI: 4788) or TLR-2 alone (MFI: 7710) or unstimulated controls (MFI: 4278); the expression of IgM lagged behind with similar treatments (MFI: 8884, 5877, 6410, 4040 on treatment with TLR2.CD40, CD40, TLR-2 and unstimulated controls, respectively) (Fig. S2 B; left panel). Expression pattern of IgD and IgM revealed that IgD progressed ahead of IgM expression, when signals were delivered together (Fig. 2D). Further a reciprocal relationship was observed in the genes encoding IgD and IgM (Fig. S2 B; right panel). We found that the Igd gene expression is lower in activated B cells as compared to Igm but the protein expression is higher. These results indicated an intricate relationship in the gene and protein expression of IgD and IgM in activated B cells.

### TLR2.CD40 signaling enhances calcium flux, ERK and Akt in RB cells

We also demonstrated that RB cells stimulated via TLR2.CD40 displayed significantly enhanced fluorescence of Fluo-4 AM dye, indicating augmented calcium flux (Fig. 2E). Further, we observed substantial increase in phosphorylation of kinases such as ERK1/2 (Fig. 2F) and Akt (Fig. 2G). This also provides clue that TLR2.CD40 signaling effectively recruit the adaptor proteins responsible for generating signaling events necessary for phosphorylation and subsequent activation of cells. Overall, these results are representative of a gross functional enhancement in the activation status of RB cells and the difference observed is statistically significant, as compared to controls.

### Signaling through CD40 drives TLR-2 stimulated RB cell differentiation to marginal zone precursors

**Figure 3:**
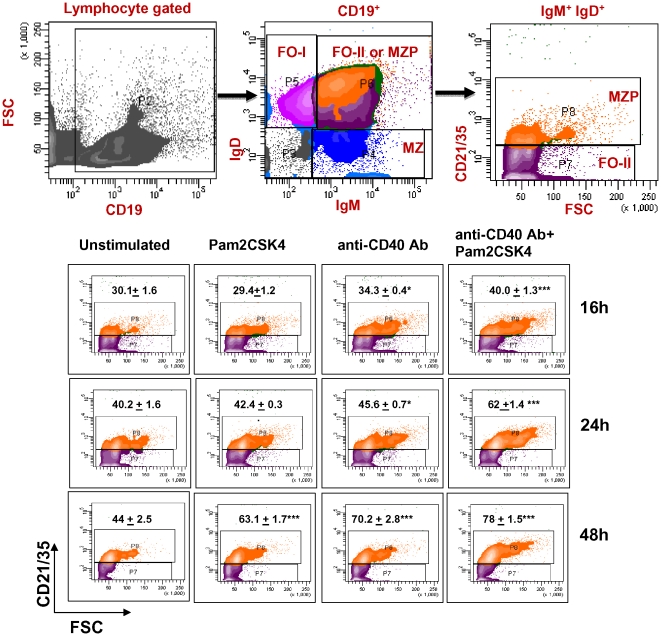
Signaling through TLR2.CD40 differentiates RB cells into marginal zone precursors. Signaling was delivered in RB cells with Pam2CSK4 and anti-CD40 Ab for 16 h–48 h. Upper panel shows the gating scheme for defining different B cell subsets: FO-I (CD19+ IgDhi), FO-II (CD19+ IgDhi IgMhi), MZP (CD19+ IgMhi IgDhi CD21/35hi) and MZ (CD19+ IgMhi). Lower panel shows contour diagrams of MZP in stimulated B cells at different time durations. Values indicate the average change (mean ± SEM) in percent population of CD21/35hi expressing B cells. Contours represent one of the three independent experiments. ‘*’, ‘**’, ‘***’ indicates p<0.05, p<0.01, p<0.001 respectively.

To check the influence of TLR2.CD40 signaling on an early differentiation of RB cells into long-lived and short-lived follicular cells (FO-I, FO-II), marginal zone precursors (MZP) and marginal zone (MZ) cells, we monitored the expression of CD19, IgD, IgM and CD21/35 (Fig. 3, upper panel). Intriguingly, we found an early (16 h) differentiation of MZP, which continued till 48 h (Fig. 3, lower panel). The percentage of MZP cells enhanced significantly (16 h: 40%; 24 h: 62%; 48 h: 78%) for all the time periods. At any given time point, this was significantly higher than unstimulated cells. Such augmented differentiation was not seen in other B cell subsets like FO-I and FO-II. This observation holds significance because MZ cells serve to bridge the innate and adaptive immune response due to their capability to respond to foreign Ags more rapidly than follicular B cells [35]. These results further corroborated well with the gene expression data where we noted significant upregulation in the genes responsible for B cell activation and differentiation. We noticed upregulation in the intensity of Stat5 gene responsible for B cell differentiation and IgG gene rearrangement downstream of IL-7R. It also augmented the display of positive regulators of B cell differentiation such as Cxcr5 (Chemokine receptor 5), Hdac9 (Histone deacetylase related protein), Gpr183 (G protein coupled receptor 183), Il2rg (IL-2R gamma), Adam17 (ADAM metallopeptidase domain 17). TLR2.CD40 triggering also lead to downregulation of negative regulators of differentiation such as Bad, Bcl2, Xrcc4, Inpp5d etc. (Table S3).Thus, these results clearly indicated that synchronized signaling through TLR2.CD40 enhances the differentiation of RB cells into marginal zone precursors and promote their developmental process.

### Costimulation through CD40 enhances isotype secretion, class switch recombination and AID expression in TLR-2 stimulated RB cells

**Figure 4:**
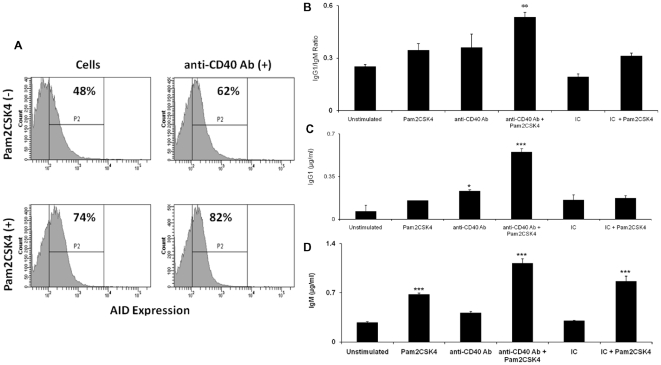
CD40 costimulation of TLR-2 elicited RB cells augments the expression of AID, class switch recombination and IgG1/IgM secretion. Signaling through TLR-2 and CD40 was delivered with Pam2CSK4 and anti-CD40 Ab respectively in RB cells. (A) Cells were stained for expression of intracellular AID after 48 h of stimulation and data were analysed by flowcytometry. Values represent the percentage of cells expressing AID. Histograms are representative of three different experiments. (B) Ratio of IgG1/IgM (index of CSR) was calculated on the basis of their surface expression after 48 h of stimulation. Data are pooled from three independent experiments and expressed as mean ± SD. (C) Secretion of IgM and IgG1 was estimated by sandwich ELISA in the culture SNs after stimulating cells for 7 d. Data are expressed as mean ± SEM from 2–3 independent experiments. ‘*’, ‘**’, ‘***’ indicates p<0.05, p<0.01, p<0.001 respectively.

It has been well documented that activation induced deaminase (AID) is involved in somatic hypermutation (SHM), class switch recombination (CSR) and somatic hyperconversion (SHC) in germinal centre B cells [36]–[38]. Recently, it has also been reported that AID activity is not just restricted to mature B cells; rather it also acts on developing B cells. Moreover, the expression of AID in B cells is not dependent on T cells but it can be modulated by BCR and TLRs [39], [40]. These findings intrigued us to monitor whether concerted triggering through TLR2.CD40 modulate AID expression and enhance CSR and isotype secretion. We found increased percentage of RB cells expressing AID (Fig. 4A) and these cells exhibited an enhancement in IgG1/IgM surface expression ratio (Fig. 4B) and secreted elevated levels of IgM and IgG1 (Fig. 4C,D). These data further authenticate the concept that signaling through TLR-2 and CD40 can influence RB cells by promoting CSR by expanding the percentage of AID^+^ cells.

### TLR2.CD40 signaling leads to enhanced antigen uptake ability of RB cells

**Figure 5:**
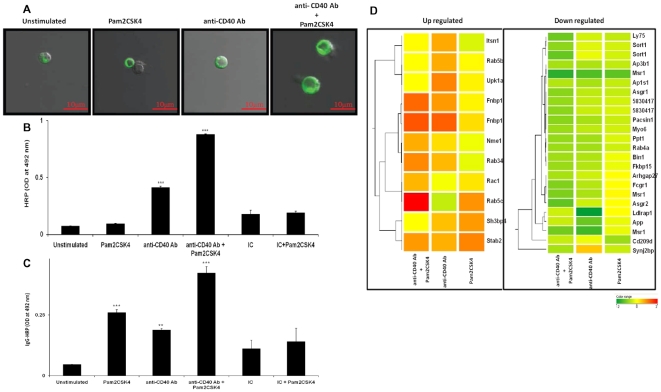
TLR-2 stimulated RB cells exhibits enhanced fluid phase pinocytosis and receptor mediated endocytosis on CD40 costimulation. RB cells were stimulated with Pam2CSK4 and anti-CD40 Ab for 16 h. (A) For confocal studies, cells were pulsed with BSA-FITC and the extent of antigen uptake is depicted through ‘Z’ stacks of stimulated B cells with respect to controls. Pictures shown here are representative of three independent experiments. Scale bar: 10 µm. (B, C) Stimulated B cells were pulsed with soluble HRP (B) or IgG-HRP (C) after stimulation and cells were washed and lysed. HRP uptake was measured colorimetrically. Values were normalized using experimental blanks and control cells which were maintained on ice. The data represents mean ± SEM of three individual experiments. (D) Dendrograms showing modulations in the expression of genes involved in endocytosis. Genes showing upregulation (upper panel) and downregulation (lower panel) were plotted with respect to control (unstimulated; assigned value ‘0’). P values are indicated in the text. Scale bar: −2 to +2. ‘*’, ‘**’, ‘***’ indicates p<0.05, p<0.01, p<0.001 respectively.

Next, we monitored whether triggering through TLR2.CD40 can influence the antigen uptake capability of B cells. Interestingly, the confocal microscopy results demonstrated substantial improvement in antigen uptake by B cells (Fig. 5A). Further, we did a quantitative enzyme based colorimetric assay with soluble HRP as an Ag. Concordant to the above results, HRP content was significantly better in the B cells stimulated through TLR2.CD40 than controls (Fig. 5B). To demonstrate receptor-mediated endocytosis, we used anti-mouse IgG-HRP Ab as an Ag to target HRP through BCR. As expected, we observed enhanced uptake of HRP (Fig. 5C). These findings signify that costimulation through CD40 of TLR-2 primed RB cells enhances the antigen uptake ability through both pinocytosis as well as receptor mediated endocytosis.

We further supported these findings by microarray experiments (Fig. 5D). Microarray analysis exhibited a profound increase in the expression of Fnbp1 (p = 0.0004), Nme1 (p = 0.0003), Rab34 (p = 0.0021), Rab 5c (p = 0.00001) and Stab2 (p = 0.06) genes (Fig. 5D, left panel). These genes play a crucial role in antigen internalization and endocytosis [41]–[44]. Some of the receptor proteins such as Msr1 (macrophage scavenger receptor1) and Fcgr (Fc gamma receptors) were downregulated in such B cells (Fig. 5D, right panel). This decline was, however, balanced by significant upregulation of other genes such as Rab, thereby shifting the equilibrium towards enhanced endocytosis.

### TLR-2 stimulated RB cells acquire enhanced ability to help T cells on CD40 costimulation

**Figure 6:**
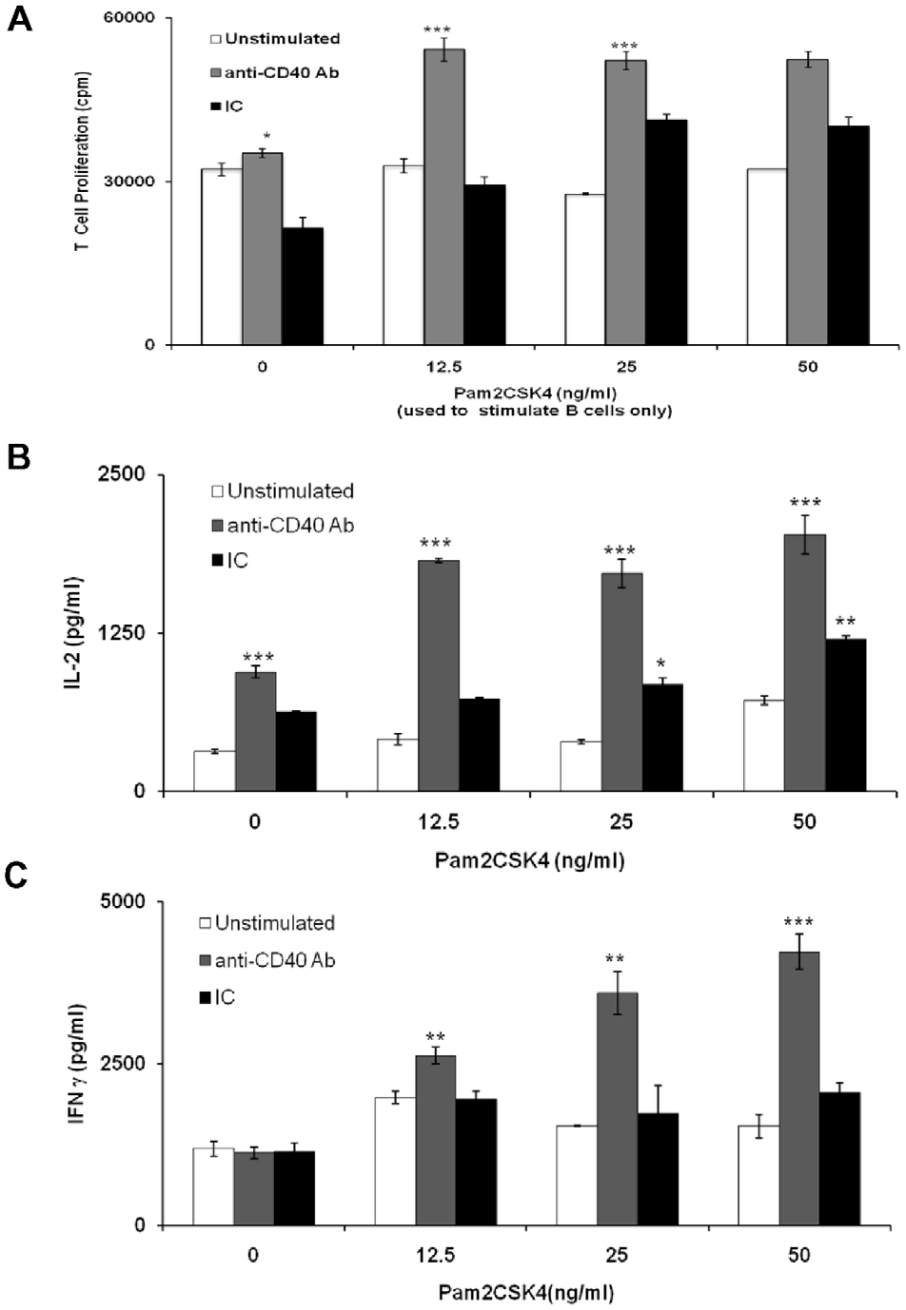
TLR-2 stimulated RB cells acquire enhanced ability to help T cells on CD40 costimulation. Signaling in B cells was delivered through TLR-2 and CD40 using Pam2CSK4 and anti-CD40 Ab respectively for 16 h. After stimulation, B cells were irradiated and co-cultured with allogenic CD4 T cells. (A) After 48 h, 3H-thymidine was added in the cultures and proliferation was checked 16 h later by ß-scintillation counting. (B, C) IL-2 was estimated 24 h later and IFN-γ after 48 h in the supernatants of parallel co-cultures. Data expressed as mean ± SEM of triplicate wells are representative of two independent experiments.

Next we addressed was whether TLR2.CD40 stimulated B cells acquired enhanced ability to stimulate CD4 T cells. Significantly (p<0.001) better proliferation, IL-2 and IFN-γ release was observed from the T cells co-cultured with TLR2.CD40 stimulated B cells (Fig. 6 A, B, C). Further, these CD4 T cells displayed activation phenotype as evidenced by the expression of CD25hi, CD44hi, CD62Llo and CD69hi (Fig. S3,A). Moreover, a closer look at the genes profiling responsible in T cell activation revealed an augmented expression of Adora (adenosine receptor needed for adenylyl cyclise activation), Il2ra (IL-2 receptor), Prkcq (calcium dependent T cell activator) and downregulated Malt1 (for Bcl-2 mediated NF κB activation of lymphocytes) (Fig. S3, B). This indicated that TLR2.CD40 stimulated B cells exhibited enhancement in the expression of receptors and mediators responsible for T cell activation.

## Discussion

Primary immune response is initiated by cognate interactions between T cells and APCs like B cells, DCs and macrophages, resulting in bidirectional signaling that modulate the function of both the cells [6], [8], [21], [45]. Various soluble mediators and cell surface components direct this process. Elucidating role of molecules involved in regulation of cell-cell interactions is crucial for understanding the immunological processes and for improving therapeutic strategies. Conventionally, role of BCR, IL-4 and CD40 have been implicated in the initiation of sequence of events leading to proliferation, activation and differentiation of B cells. Binding of antigen to BCR relays survival signals and prevents apoptosis. This interaction is also necessary to maintain optimal pool of B cells in the peripheral circulation. Besides this classical paradigm, evidences have started surfacing where B cells have been shown to be modulated independent of BCR. For example, signaling B cells through CD86 enhances their survival, proliferation and differentiation [6], [21], [46]. This poses a plausible existence of an alternative mechanism where B cells can be stimulated in a BCR independent manner.

Based on the above mentioned information, the present study was designed to elucidate the alternative pathway of B cell activation by engaging costimulatory molecules and TLRs. In particular, we focused on costimulatory effect of CD40 on TLR-2 stimulated RB cells in bolstering their activation, maturation and differentiation. Following six major findings have emerged in context of B cells from this study (i) improvement in proliferation and modulation of the expression of related genes; (ii) upregulation of activation and maturation markers; (iii) enhanced calcium flux and phosphorylation of ERK1/2 and Akt; (iv) increased ability to pinocytose and endocytose antigen; (v) induction in class switch recombination and enhanced immunoglobulin secretion; (vi) improved ability to activate T cells.

The results of present study support the fact that B cells can be activated not only through conventional pathway; but also through alternative route employing TLRs and costimulatory molecules [6], [8], [21], [45], [46]. Further, it also indicates that CD40 can effectively induce the activation, proliferation and differentiation of RB cells that have received first signal via TLR-2. We resolutely indicate that TLR2.CD40 stimulated RB cells upregulates the expression of activation markers and also promote their maturation. Such stimulated B cells preferentially differentiate into marginal zone precursors, which are considered as “innate B cells” and may aid in bridging innate and adaptive immunity. Interestingly, TLR-2 stimulated RB cells on CD40 costimulation acquire significantly improved ability to engulf antigen through both pinocytosis and receptor-mediated endocytosis and they acquire enhanced capacity to help T cells; ensuing robust T cell activation. This observation may have important implication since it can help in designing vaccination strategies that can reduce the time threshold of immune response required for activation of B cells; thereby can clear the pathogens in shorter duration.

Although RB cells express basal level of costimulatory molecules, yet their role has been implicated to act as APCs that can present antigen to T cells and thereby induces their activation [47]. Moreover, it has been discussed that for optimal activation of cells, it is not necessary to have a dramatic difference in the levels of costimulatory molecules [21]. We support this and argue that not too much difference in the modulation of activation parameters is needed by the cells to be functionally and physiologically active, as evidenced by our data. In fact, strong stimulation may lead to the activation of autoreactive T cells. Hence we very carefully titrated the dose of both anti-CD40 Ab and Pam2CSK4, and used suboptimal levels to combine their effects.

Further, it has been decisively demonstrated that there exists a phenomenon of bidirectional costimulation wherein B cells are also activated through signals emanating from B cell-T cell interaction [8], [21]. Hence these findings suggest that apart from BCR, there are other molecules which deliver positive signals for B cell survival, proliferation and differentiation. We extrapolate these findings and implicate new molecules that can further support this phenomenon of bidirectional costimulation. CD40 is considered to be an important molecule affecting B cell biology. On the other hand, unlike TLR-4, the role of TLR-2 has not been much elucidated specifically on RB cells. However, it has been implied that TLR-2 deficiency impairs humoral immune responses in case of bacterial infections [17]. We propose that there exists an alternative pathway of BCR-independent signaling which can have important implications in B cell biology. Whether this pathway employs different downstream signaling adaptor molecules or bypasses some of the downstream signaling molecules, are some of the questions to be addressed further. Moreover, it seems interesting to understand if BCR signaling can add to the effectiveness of these signals.

In essence, we demonstrate very precisely in this study, how adaptive signals originating from CD40 costimulatory molecules and innate immune signals emerging from TLR-2 can synergistically bolster the activity of RB cells. This further reinforces the belief that molecules of innate and adaptive immunity partner with each other in bridging these two arms of immunity; thereby facilitating faster and better B cell response. In conclusion, this study may help in designing vaccines and immunotherapies involving CD40 and TLR-2 that can significantly boost B cell response.

## Materials and Methods

### Mice

BALB/c, C57BL/6 and C3He mice (male or female, 4–6 weeks) were procured from National Institute of Pharmacological Education and Research (NIPER, Mohali), National Institute of Immunology (NII, New Delhi) and National Institute of Nutrition (NIN, Hyderabad), India.

### Ethics statement

All experiments were approved by the Institutional Animal Ethics Committee of Institute of Microbial Technology and performed according to the National Regulatory Guideline issued by Committee for the Purpose of Supervision of Experiments on Animals (No. 55/1999/CPCSEA), Ministry of Environment and forest, Govt. of India.

### Reagents

Resting B cells isolated from mouse splenocytes were cultured in RPMI-1640 medium supplemented with FBS (10%), L-glutamine (2 mM), penicillin (50 µg/ml), streptomycin (50 µg/ml), HEPES (2.38 mg/ml), sodium bicarbonate (2.2 mg/ml) and 2-mercaptoethanol (0.05 mM) under standard conditions, as described elsewhere [8]. B cell and CD4 T cell enrichment cocktails, biotin-conjugated anti-mouse CD43 (S7), CD45R (B220, RA3-6B2), CD40 (3/23), isotype control (IgG2aκ,R35-95), MHC class I (H-2D[d], 34-2-12), MHC class II (IA^d^, AMS-32.1) and CD23 (B3B4); FITC conjugated CD21/35 (7G6), CD25 (3C7), CD80 (16-10A1); PE-labeled anti-mouse IgD (11-26c.2a), CD69 (H1.2F3), CD8a (53-6.7), CD86 (GL1); PE-Cy5 conjugated CD5 (53-7.3) CD44 (IM7); PE-Cy7 coupled IgM (R6-60.2), APC or PE-Cy5 conjugated streptavidin; APC labeled CD62L (MEL-14), APC-Cy7 linked CD19 (1D3) and Pacific blue tagged CD45R (B220, RA3-6B2), phosflow antibodies and reagents were purchased from BD Biosciences (San Diego, USA). Alexa-647 coupled anti-mouse TLR-2 (T2.5) and FITC coupled anti- mouse AID (eBio911) was procured from eBiosciences (San Diego, USA). All ELISA reagents were purchased from BD Biosciences. All other standard reagents were procured from Sigma unless otherwise mentioned. TLR-2 ligand (Pam2CSK4) was obtained from Invivogen (San Diego, USA). Fluo4-AM dye and pluronic acid were procured from Invitrogen Molecular Probes (Carlsbad, California 92008).

### B cell isolation and stimulation

Resting B cells (RB) were isolated from mice splenocytes using well established negative selection method. Briefly, single cell suspensions of splenocytes were prepared and treated with B cell enrichment cocktail supplemented with biotin-anti-CD43 Abs. The cells were then treated with same volume of streptavidin-magnetic beads and negatively selected on BD IMagnet. The purity of RB cells obtained was >98% as depicted by flow cytometry (CD45R^+^CD19^+^CD43^−^CD4^−^CD8^−^). Purified RB cells were incubated with biotin conjugated anti-CD40 and isotype control Abs (IgG2a,κ) (0.5 µg/10^6^ cells) for 30 min on ice followed by cross-linking with equivalent concentration of streptavidin, under similar conditions. The cells were washed and then plated in culture plates containing either medium alone or medium containing Pam2CSK4. Suitable controls such as cells alone, cells stimulated with anti-CD40 Abs or isotype matched Abs, with streptavidin or with Pam2CSK4 alone were also kept in all experiments.

### B cell proliferation

Anti-CD40 Ab (0.5 µg/10^6^ cells) stimulated RB cells (5×10^4^ cells/well) were incubated with different concentrations of Pam2CSK4 (0–100 ng/ml) in 96 well U-bottom plates for different time durations. Cells incubated with isotype matched control Ab, Pam2CSK4, streptavidin and medium alone were used as controls. After each stipulated time (4 h–48 h), radioactive thymidine (0.5 µCi/well) (Amersham, Buckinghamshire, UK) was incorporated in the cultures and incubated for 16 h. Later, plates were harvested onto glass-fibre filter mats using a Tomtec-Harvester-96 (Tomtec, Hamden, CT). Proliferation was measured in terms of radioactive thymidine incorporation (counts per minute, cpm) using ß-scintillation counter (Wallac-1450 Microbeta Trilux, Perkin Elmer, Waltham, MA).

### Flow cytometry analysis of B cell activation, maturation and differentiation

RB cells (5×10^4^/well) were stimulated with anti-CD40 Ab (0.5 µg/10^6^ cells) with or without Pam2CSK4 (50 ng/ml), as described, for specific durations (16 h–48 h). Cells were stained for molecules that define B cell activation, differentiation and maturation. Briefly, cells were harvested, washed and stained with anti-mouse fluorochrome labeled anti-CD80, CD86, CD40, CD21/35, CD5, IgD, IgM, CD93, CD19, TLR-2 Abs and their respective isotype matched controls for 30 min at 4°C. When staining with biotinylated Abs, cells were incubated with biotin conjugated Abs (CD23, MHC class I, MHC class II) for 30 min at 4°C followed by incubation with secondary reagents (streptavidin-PE-Cy5/APC). Finally, cells were washed and fixed in 1% paraformaldehyde. The flow cytometry data were acquired using FACS Calibur and FACS Aria II (BD Biosciences, San Jose, CA). Data were analyzed by DIVA software (BD Biosciences, San Jose, CA).

### Calcium flux assay

TLR2.CD40 stimulated RB cells were loaded with Fluo-4 AM dye (5 µM) and pluronic acid (0.02%) in RPMI at 37°C for 30 min in dark in serum free media and washed extensively. The cells were kept for 30 min dark at RT and the relative fluorescence was analysed by flow cytometry. In each sample, unstimulated controls were acquired for the first 60 seconds for baseline acquisition, and subsequently stimulated samples were acquired (total acquisition per sample was done for 10 min).

### Phosflow analysis

RB cells were stimulated for 10 min at 37°C and cells were stained for Phosflow analysis using manufacturer's instructions (BD Biosciences). Briefly, cells were fixed with pre-warmed lyse fix buffer, washed, permeabilized and incubated with anti-ERK ½ or anti-Akt antibodies for 30 min at RT in dark. Thereafter the cells were washed again and analysed using BD FACS Calibur.

### Antigen uptake assay

RB cells (2×10^5^/well) were stimulated with anti-CD40 Ab (0.5 µg/10^6^ cells) in the presence or absence of Pam2CSK4 (50 ng/ml) for 16 h. The cells were harvested, washed and then pulsed with either free HRP (200 µg/ml) or anti-mouse IgG-HRP Ab (1∶100). Cells were incubated on ice for 15 min followed by incubation in RPMI-FBS-5% at 37°C. Antigen was chased for 30–60 min and uptake was arrested by adding chilled PBS and by transferring cells on ice. Cells were washed extensively with ice cold PBS-FBS-1% and then treated with pronase and washed once again. The cells were lysed using 10 mM Tris-HCl and 0.05% Triton X-100 for 30 min on ice, with intermittent vortexing. Intracellular HRP was estimated colorimetrically in the cell lysates using OPD-H_2_O_2_ chromogen-substrate system. Cells maintained at 4°C, undigested with pronase and unlysed cells were kept as controls. HRP activity in test samples was suitably normalized with controls. For confocal analysis, RB cells (2×10^5^/well) were stimulated as mentioned above. The cells were pulsed with soluble BSA-FITC (50 µg/ml) and chased for 30 min. The cells were washed extensively (4×) with ice cold PBS and fixed with 4% paraformaldehyde. The cells were washed and placed on poly-L-lysine coated cover slips and imaged using Nikon A1 Confocal Laser Microscope system. Z-stacks were taken and extent of antigen internalization was monitored among experimental and control samples. Analysis was done using Nikon NIS-C image analysis software.

### B cell help to T cells

RB cells (10^5^/well) obtained from BALB/c were stimulated with anti-CD40 Ab (0.5 µg/10^6^ cells) and different concentrations of Pam2CSK4 (0–100 ng/ml) for 16 h. The cells were harvested, washed and gamma irradiated at 3000R and co-cultured with MACS purified allogenic CD4 T cells (2×10^5^/well) isolated from C3He mice. Parallel cultures were set for T cell proliferation, cytokines and activation markers. T cell proliferation was determined by incorporation of ^3^H-thymidine (0.5 µCi/well) after 48 h and was expressed in terms of radioactive thymidine incorporation (counts per minute, cpm). For cytokine estimation, supernatants from the experimental and control wells were collected after 24 h for IL-2 and 48 h for IL-4 and IFN-γ. Cytokines were estimated by sandwich ELISA, following manufacturer's instructions. The levels of cytokines were calculated using serial dilutions (log_2_) of standard recombinant cytokines and expressed as pg/ml. T cell activation markers were studied by flow cytometry. Cells were harvested after 48 h, washed and stained for the expression of CD25, CD69, CD44 and CD62L with their respective Abs. Finally, cells were washed and fixed in 1% paraformaldehyde and analysed by flow cytometry.

### Isotype ELISA

RB cells (10^5^/well) were stimulated with anti-CD40 Abs (0.5 µg/10^6^ cells) with or without Pam2CSK4 (50 ng/ml). Supernatants (SN) were collected after 5–7 days and secretion of IgM and IgG1 was determined by standard sandwich ELISA. Briefly, SNs were added on anti-IgM or anti-IgG1 Abs coated plates. IgM and IgG1 were captured by secondary biotinylated anti-mouse IgM or IgG1 Abs, respectively, followed by avidin-HRP/OPD-H_2_O_2_ for colorimetric estimation. Results expressed as µg/ml of isotype antibody secreted were calculated using serial dilutions (log_2_) of standard IgM and IgG1 for reference curves.

### PI-Annexin Assay

RB cells (5×10^4^/well) were stimulated with anti-CD40 Ab (0.5 µg/106 cells) in the presence and absence of Pam2CSK4 (50 ng/ml) for 48 h at 37°C in 200 µl of RPMI-FBS-10%. The cells were harvested, washed and resuspended in binding buffer [0.01 M HEPES (pH 7.4), 0.14 M NaCl, 2.5 mM CaCl2]. FITC conjugated Annexin V antibody (5 µl per tube) and 5 µl of propidium iodide (50 µg/ml) were added to the cells. The cells were incubated in dark for 15 min at RT. Thereafter, binding buffer (400 µl) was added and cells were acquired immediately using BD FACS Calibur flowcytometer.

### Intracellular AID staining

Resting B cells (2×10^5^/well) were stimulated with anti-CD40 Ab in the presence and absence of Pam2CSK4 (50 ng/ml) for 48 h at 37°C in 200 µl of RPMI-FBS-10%. The cells were harvested, washed and surface staining was done for IgG1 and IgM. The cells were washed and fixed with 4% paraformaldehyde. The cells were gently vortexed, washed and permeabilized with 1 ml of IX permeabilization buffer for 30 minutes at 4°C. Anti-mouse AID-FITC antibody was added and incubated for 4 hours in dark at 4°C. Finally the cells were washed with FACS buffer and AID expression was analysed by flow cytometry.

### RNA isolation

RB cells (1.5×10^7^ cells/combination) were stimulated with and without anti-CD40 Abs (0.5 µg/10^6^ cells) and cultured for 4 h in the presence and absence of Pam2CSK4 (50 ng/ml). Cells were harvested and washed 2× with PBS. RNA isolation was performed using Qiagen Rneasy Minikit as per the manufacturer's instructions. RNA concentration and purity was determined at an optical density ratio of 260/280 using the Nanodrop® ND-1000 spectrophotometer (NanoDrop Technologies, Wilmington, DE) and the integrity of total RNA was verified on an Agilent 2100 Bioanalyzer using the RNA 6000 Nano LabChip (Agilent Technologies). RNA was stored at −80°C until use.

### Microarray analysis

Microarray was performed by Genotypic Technology Pvt. Ltd. (www.genotypic.co.in). The samples for gene expression were labeled using Agilent Quick-Amp labelling kit (p/n5190-0442). The labeled cRNA samples were hybridized on to a Genotypic designed Custom Whole Genome Mouse 8×60k (AMADID No: 26986). 800 ng of Cy3-labeled samples were fragmented and hybridized. Fragmentation of labeled cRNA and hybridization were done using the gene expression hybridization kit of Agilent. Hybridization was carried out in Agilent's Surehyb Chambers at 65°C for 16 h. The hybridized slides were washed using Agilent gene expression wash buffers and scanned using the Agilent Microarray Scanner G2505C at 3 µ resolution. Data extraction from images was done using Feature Extraction Software v 10.5.1.1 of Agilent. Feature extracted data were analyzed using GeneSpring GX v 11 software from Agilent. Genes were classified based on the functional category and pathways using GeneSpringGX Software and Genotypic Biointerpreter-Biological Analysis Software.

### Statistics

Data were analyzed by Student's ‘t’ test, non-parametric Mann-Whitney two tailed test and repeated measure ANOVA with post Student-Newman-Keuls multiple comparisons test by Graph Pad InStat 3 software. ‘p’ values are denoted with respect to unstimulated controls.

## Supporting Information

Figure S1 **TLR-2 stimulated RB cells upregulates the expression of costimulatory molecules MHC molecules and TLR-2 on CD40 triggering.** Signaling was delivered in RB cells with Pam2CSK4 and anti-CD40 Ab for 16 h and the expression of CD40, CD86 and CD80 was assessed by flowcytometry using respective fluorochrome conjugated Abs. Flowcytometry plots (A) are representative of one of the three experiments. The values in the inset illustrate the mean fluorescence intensity (MFI) normalized with isotype-matched control. Bar diagrams (B) represent average fold change (mean ± SEM) with respect to unstimulated controls from three independent experiments. ‘*’, ‘**’, ‘***’ indicates p<0.05, p<0.01, p<0.001 respectively. The expression of MHC-I, MHC-II and TLR-2 on TLR2.CD40 stimulated RB cell were analysed by flowcytometry (C). The values represent MFI of respective molecules normalized with isotype matched controls. Data are representative of four independent experiments.
(TIF)

Figure S2 (A) The expression of B cell activation markers CD21/35, CD23, IgD, IgM, CD5 and CD19 were analysed by multicolour flowcytometry. The values represent MFI of respective molecules normalized with isotype matched controls. Data are representative of four independent experiments. (B) Left panel indicates the simultaneous expression of IgD and IgM on RB cells when triggered through TLR2.CD40. Expression was analysed by flowcytometry and values are indicated in the main text. The right panel indicates the fold change in the expression of genes encoding IgD and IgM with respect to unstimulated controls analyzed through microarray.
(TIF)

Figure S3 (A) Signaling in B cells was delivered through TLR-2 and CD40 using Pam2CSK4 and anti-CD40 Ab respectively for 16 h. After stimulation, B cells were irradiated and co-cultured with allogenic CD4 T cells. Cells were harvested after 48 h and expression of activation markers was studied by flow cytometry using fluorochrome tagged anti-mouse CD25, CD69, CD62L and CD44 Abs. Shown here are representative contour diagrams from two independent experiments. (B) RB cells were harvested from cultures and microarray analysis was performed for modulation in expression of genes involved in T cell activation and TCR signaling. Geometric mean of the fold change in the expression of genes was calculated. Genes showing upregulation and downregulation were plotted with respect to control (unstimulated; assigned value ‘0’). Values represent geometric mean of fold change of replicate samples. ‘*’, ‘**’, ‘***’ indicates p<0.05, p<0.01, p<0.001 respectively.
(TIF)

Table S1 (A) Modulation in the expression of genes involved in B cell proliferation. RB cells were stimulated with anti-CD40 Ab and Pam2CSK4 for 4 h and RNA was isolated for microarray analysis. The different colour codes indicate degree of change in the genes expression (yellow: no change with respect to unstimulated controls; red: up regulation; green: down regulation). The values indicate geometric mean of fold change of biological replicate samples. Statistical analysis is done using One Way ANOVA and a ‘p’ value for each sample is given next to its corresponding geometric mean.
(TIF)

Table S2 **Change in the expression of gene profile involved in calcium pathway.** The table depicts modulation in the gene expression with different colour codes (yellow: no change with respect to unstimulated controls, red: up regulation, green: down regulation). The values indicate geometric mean of fold change of replicate samples. Statistical analysis was done using One Way ANOVA and ‘p’ values for each sample are given next to its corresponding geometric mean.
(TIF)

Table S3 **Change in the expression of gene profile involved in RB cell activation and differentiation.** The table depicts modulation in the gene expression with different colour codes (yellow: no change with respect to unstimulated controls, red: up regulation, green: down regulation). The values indicate geometric mean of fold change of replicate samples. Statistical analysis was done using One Way ANOVA and ‘p’ values for each sample are given next to its corresponding geometric mean.
(TIF)
