# Supplementary figures and images for: CD40 Signaling Synergizes with TLR-2 in the BCR Independent Activation of Resting B Cells
Source: PLoS One. 2011 Jun 2;6(6):e20651. doi: 10.1371/journal.pone.0020651 (PMC3107243; doi:10.1371/journal.pone.0020651)

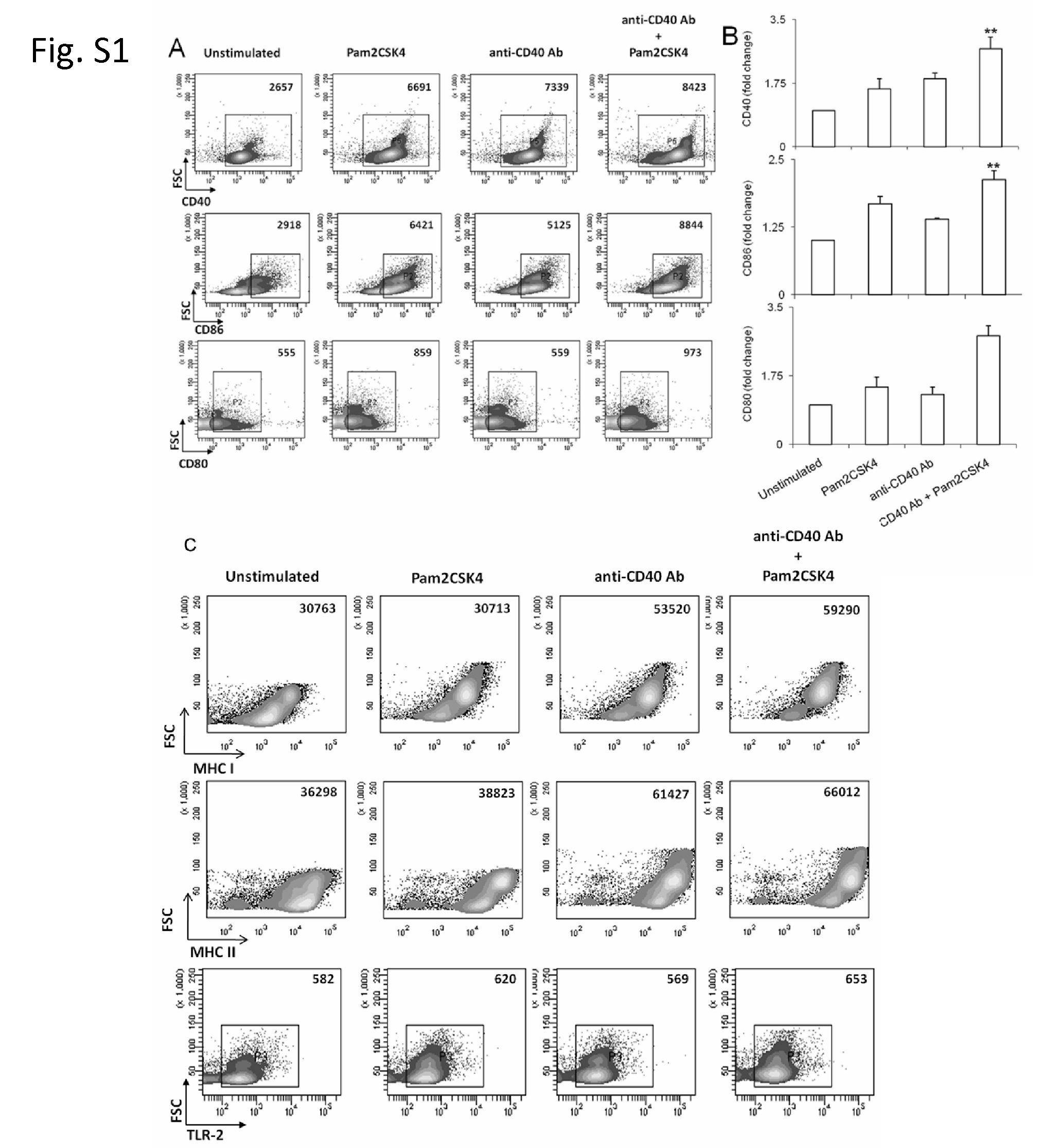

Supplement: Figure S1 — TLR-2 stimulated RB cells upregulates the expression of costimulatory molecules MHC molecules and TLR-2 on CD40 triggering. Signaling was delivered in RB cells with Pam2CSK4 and anti-CD40 Ab for 16 h and the expression of CD40, CD86 and CD80 was assessed by flowcytometry using respective fluorochrome conjugated Abs. Flowcytometry plots (A) are representative of one of the three experiments. The values in the inset illustrate the mean fluorescence intensity (MFI) normalized with isotype-matched control. Bar diagrams (B) represent average fold change (mean ± SEM) with respect to unstimulated controls from three independent experiments. ‘*’, ‘**’, ‘***’ indicates p<0.05, p<0.01, p<0.001 respectively. The expression of MHC-I, MHC-II and TLR-2 on TLR2.CD40 stimulated RB cell were analysed by flowcytometry (C). The values represent MFI of respective molecules normalized with isotype matched controls. Data are representative of four independent experiments. (TIF) [file pone.0020651.s001.tif]

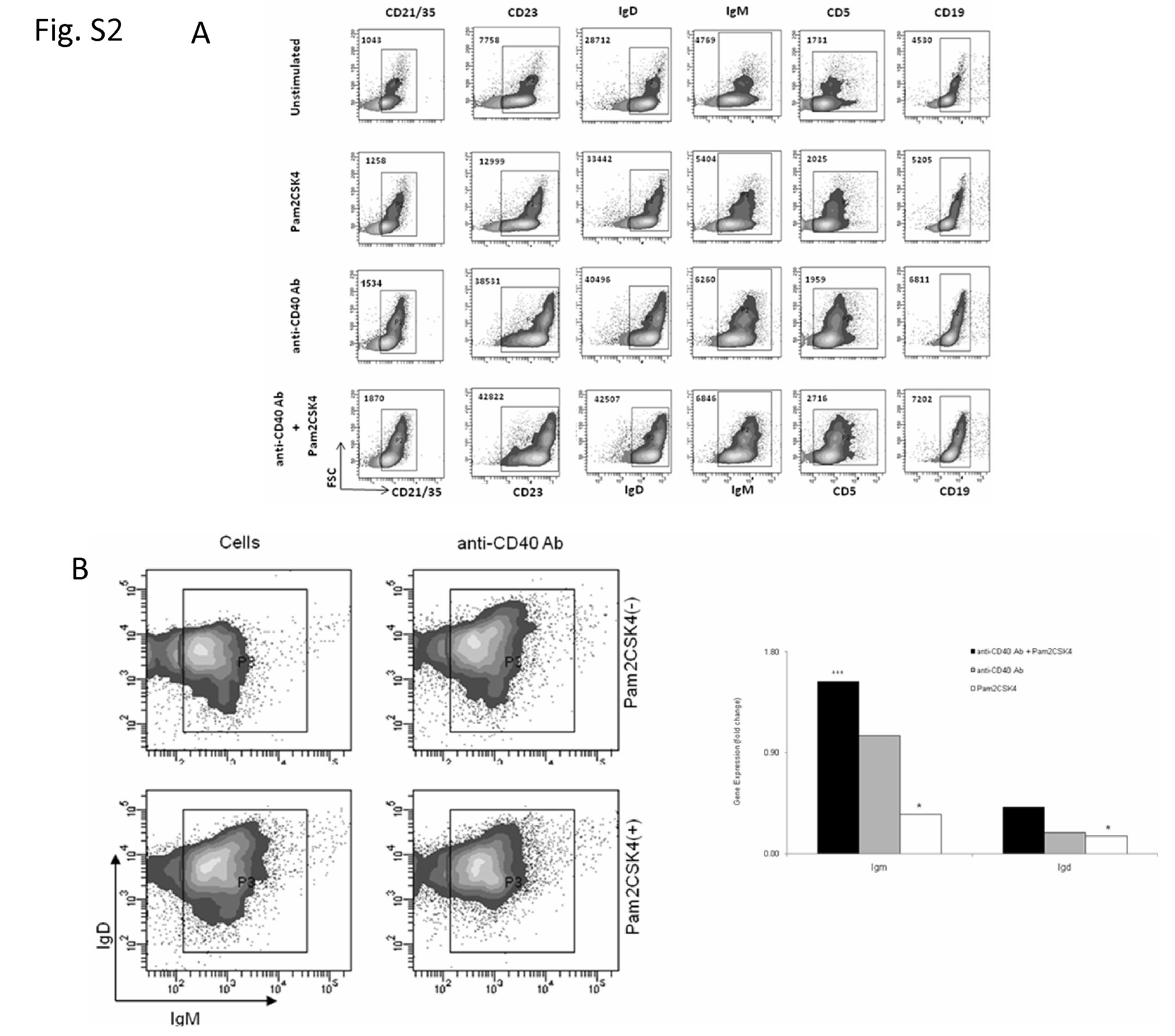

Supplement: Figure S2 — (A) The expression of B cell activation markers CD21/35, CD23, IgD, IgM, CD5 and CD19 were analysed by multicolour flowcytometry. The values represent MFI of respective molecules normalized with isotype matched controls. Data are representative of four independent experiments. (B) Left panel indicates the simultaneous expression of IgD and IgM on RB cells when triggered through TLR2.CD40. Expression was analysed by flowcytometry and values are indicated in the main text. The right panel indicates the fold change in the expression of genes encoding IgD and IgM with respect to unstimulated controls analyzed through microarray. (TIF) [file pone.0020651.s002.tif]

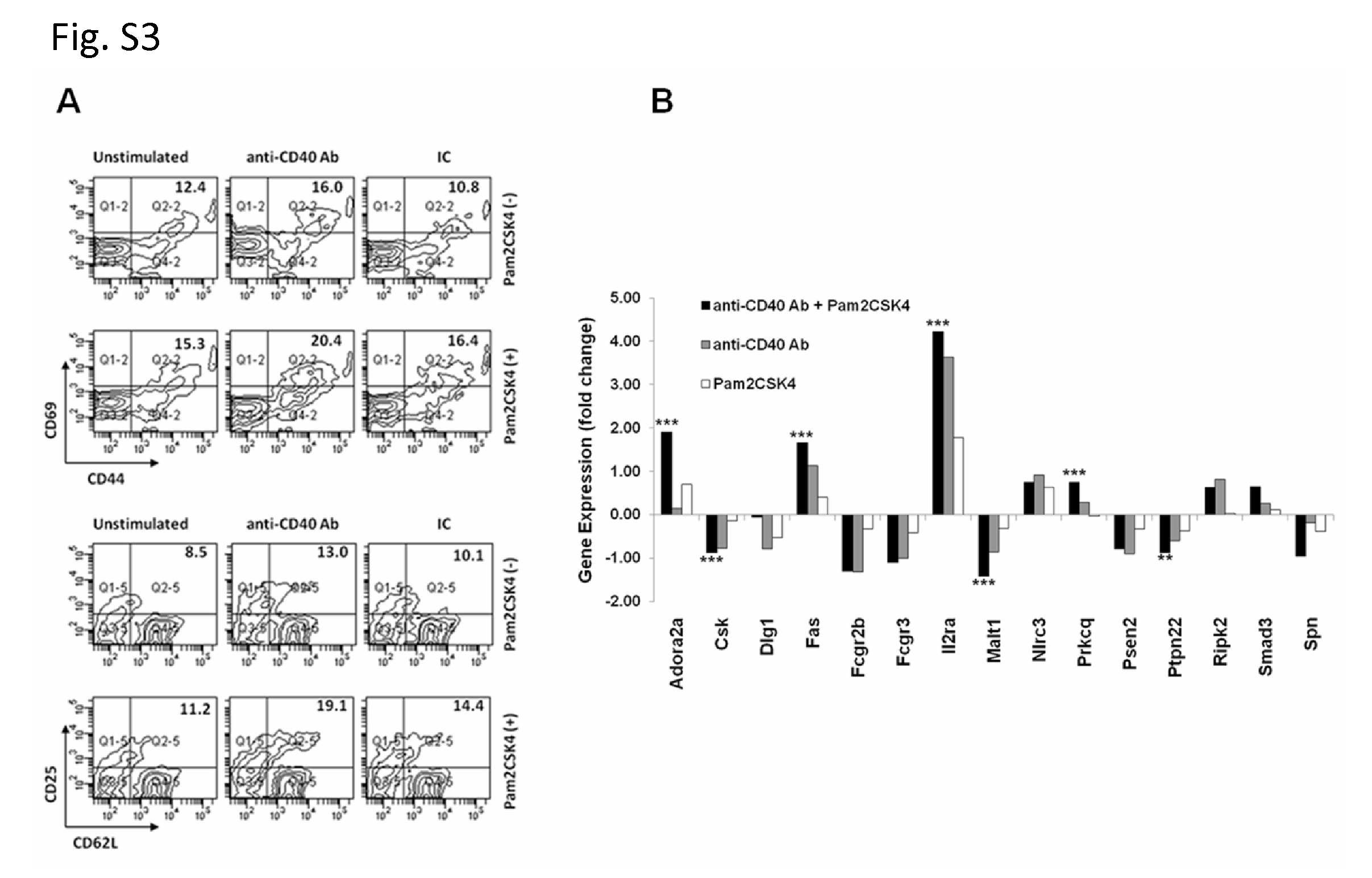

Supplement: Figure S3 — (A) Signaling in B cells was delivered through TLR-2 and CD40 using Pam2CSK4 and anti-CD40 Ab respectively for 16 h. After stimulation, B cells were irradiated and co-cultured with allogenic CD4 T cells. Cells were harvested after 48 h and expression of activation markers was studied by flow cytometry using fluorochrome tagged anti-mouse CD25, CD69, CD62L and CD44 Abs. Shown here are representative contour diagrams from two independent experiments. (B) RB cells were harvested from cultures and microarray analysis was performed for modulation in expression of genes involved in T cell activation and TCR signaling. Geometric mean of the fold change in the expression of genes was calculated. Genes showing upregulation and downregulation were plotted with respect to control (unstimulated; assigned value ‘0’). Values represent geometric mean of fold change of replicate samples. ‘*’, ‘**’, ‘***’ indicates p<0.05, p<0.01, p<0.001 respectively. (TIF) [file pone.0020651.s003.tif]

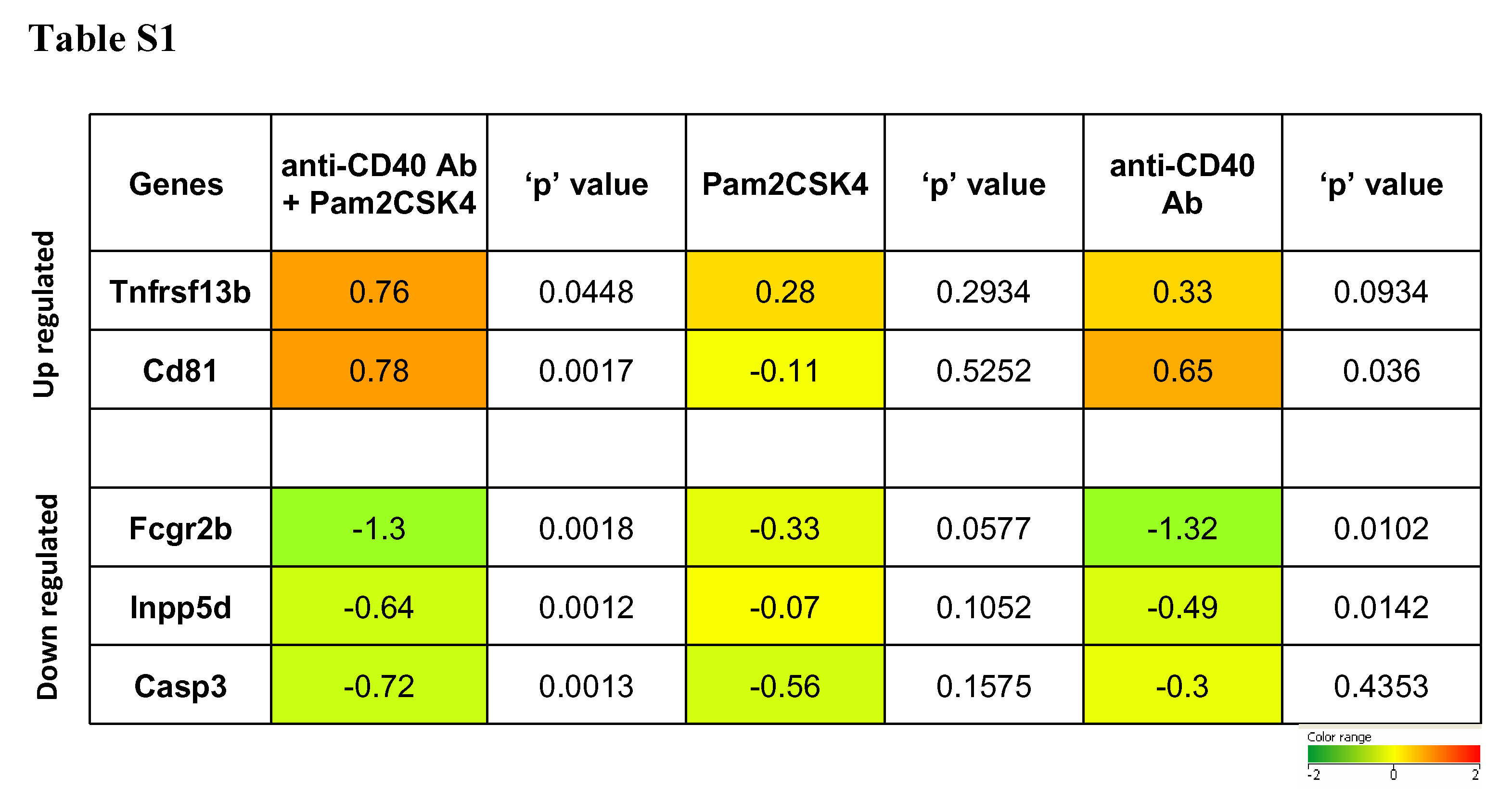

Supplement: Table S1 — (A) Modulation in the expression of genes involved in B cell proliferation. RB cells were stimulated with anti-CD40 Ab and Pam2CSK4 for 4 h and RNA was isolated for microarray analysis. The different colour codes indicate degree of change in the genes expression (yellow: no change with respect to unstimulated controls; red: up regulation; green: down regulation). The values indicate geometric mean of fold change of biological replicate samples. Statistical analysis is done using One Way ANOVA and a ‘p’ value for each sample is given next to its corresponding geometric mean. (TIF) [file pone.0020651.s004.tif]

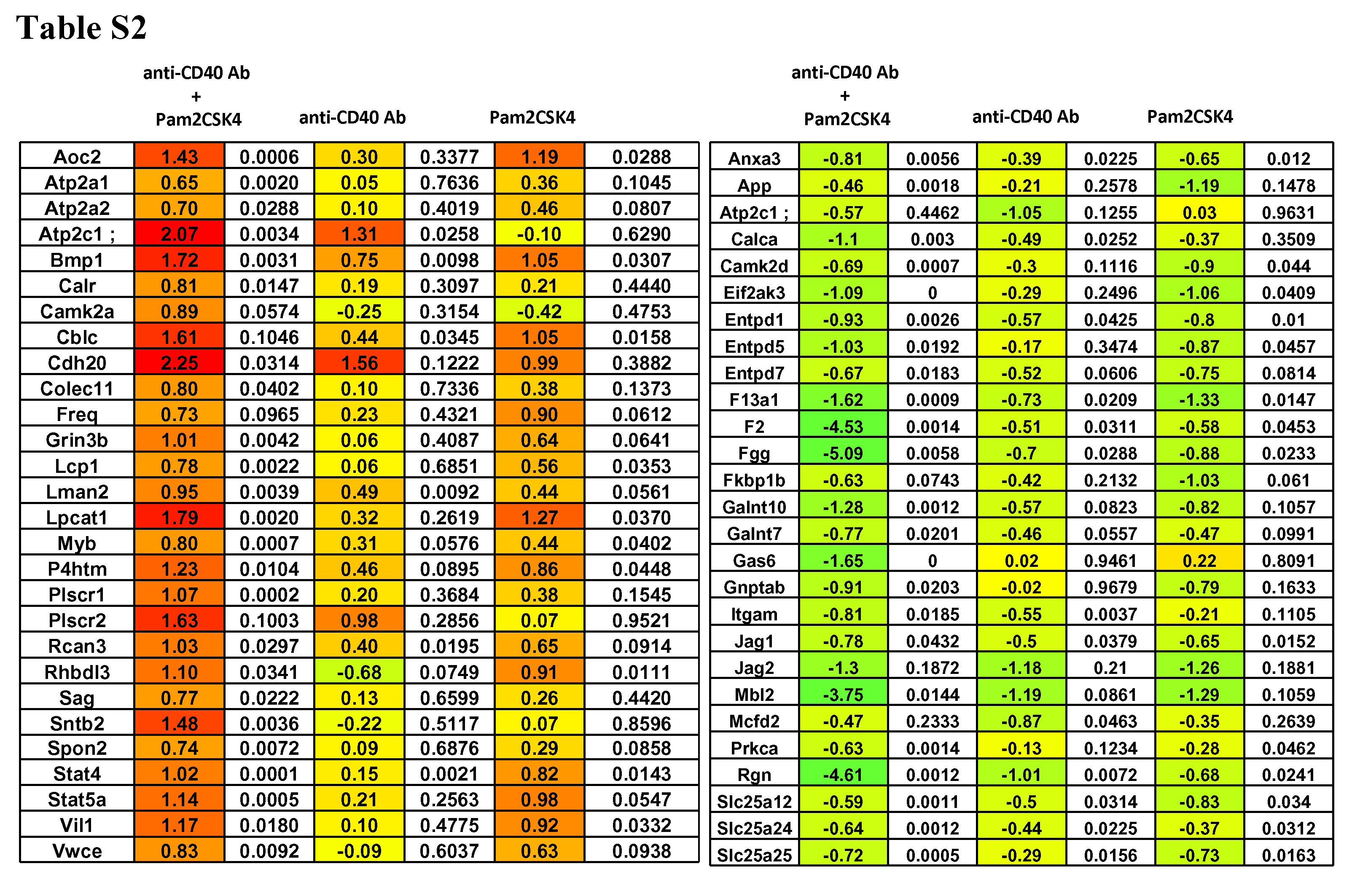

Supplement: Table S2 — Change in the expression of gene profile involved in calcium pathway. The table depicts modulation in the gene expression with different colour codes (yellow: no change with respect to unstimulated controls, red: up regulation, green: down regulation). The values indicate geometric mean of fold change of replicate samples. Statistical analysis was done using One Way ANOVA and ‘p’ values for each sample are given next to its corresponding geometric mean. (TIF) [file pone.0020651.s005.tif]

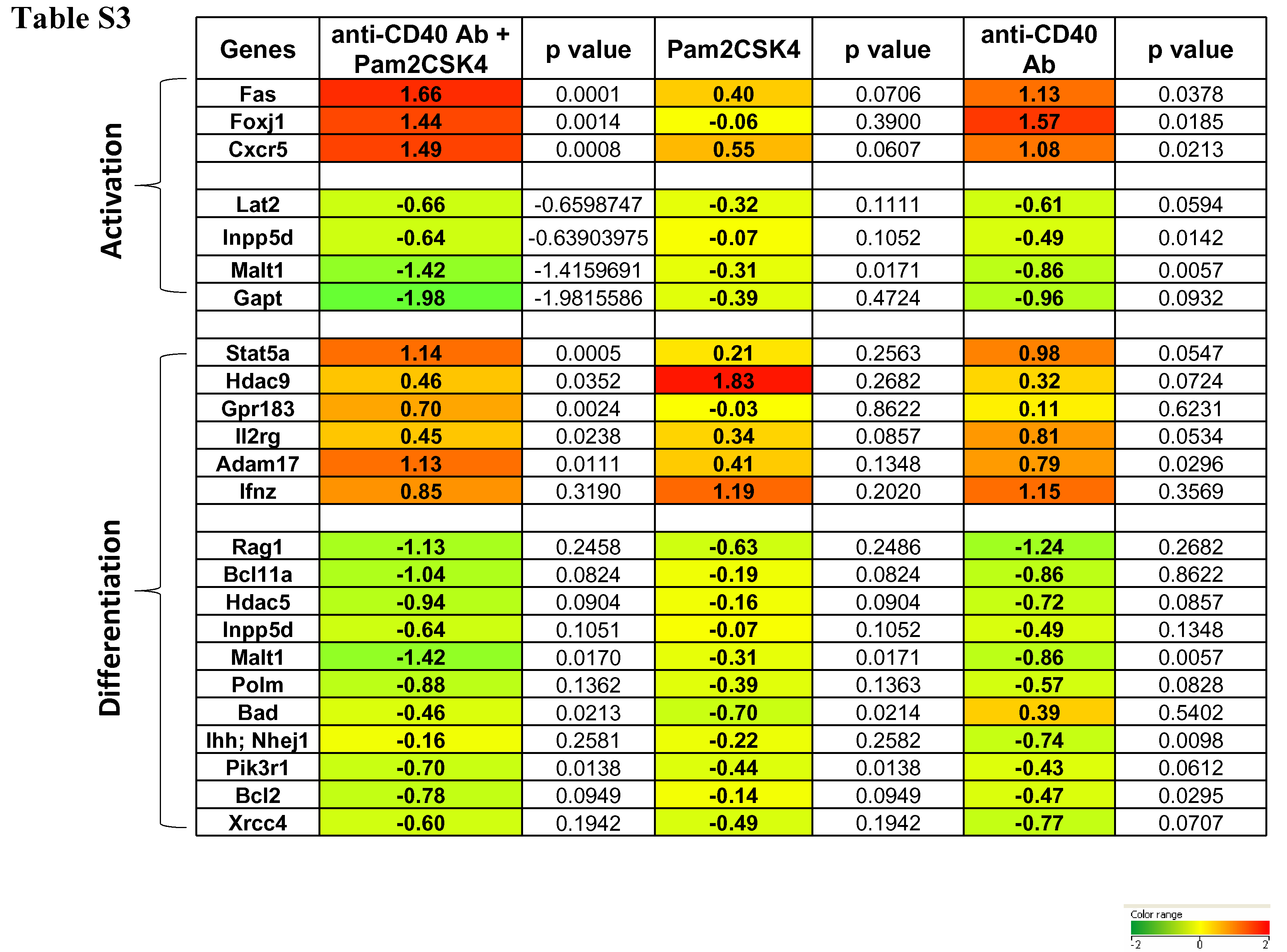

Supplement: Table S3 — Change in the expression of gene profile involved in RB cell activation and differentiation. The table depicts modulation in the gene expression with different colour codes (yellow: no change with respect to unstimulated controls, red: up regulation, green: down regulation). The values indicate geometric mean of fold change of replicate samples. Statistical analysis was done using One Way ANOVA and ‘p’ values for each sample are given next to its corresponding geometric mean. (TIF) [file pone.0020651.s006.tif]
